# Supplementary material for: Measurement matters: higher waist-to-hip ratio but not body mass index is associated with deficits in executive functions and episodic memory
Source: PeerJ. 2018 Sep 7;6:e5624. doi: 10.7717/peerj.5624 (PMC6130234; doi:10.7717/peerj.5624)
Supplement: Supplemental Information 1 [file peerj-06-5624-s001.pdf]

Table A.1. *Zero-order Correlations between Covariates and Criterion Variables*

	Episodic Memory	Executive Functions
Age	.340**	.105**
Sex	-.225**	.105**
Education	.209**	.426**
Household Income	.193**	.355**
Subjective status	.024	-.030
Hypertension	-.142**	-.203**
Diabetes	-.121**	-.145**
Stroke	-.075**	-.110**
Self-rated Health	-.180**	-.301**
Chronic disease	-.111**	-.229**
Former smoker	-.095**	-.063**
Current smoker	.024	-.042*
Alcohol	.100**	.204**
Physical exercise	.198**	.315**
Agreeableness	.049*	-.090**
Openness	.086**	.114**
Neuroticism	-.004	-.041*
Extraversion	.044*	-.037*
Conscientiousness	.101**	.077**
Perceived obesity	-.002	.025

Note. * $p < .05$, ** $p < .001$
