# Supplementary material for: Measurement matters: higher waist-to-hip ratio but not body mass index is associated with deficits in executive functions and episodic memory
Source: PeerJ. 2018 Sep 7;6:e5624. doi: 10.7717/peerj.5624 (PMC6130234; doi:10.7717/peerj.5624)
Supplement: Supplemental Information 2 [file peerj-06-5624-s002.pdf]

Appendix B: Scatterplot of the relationships between obesity and cognitive functions

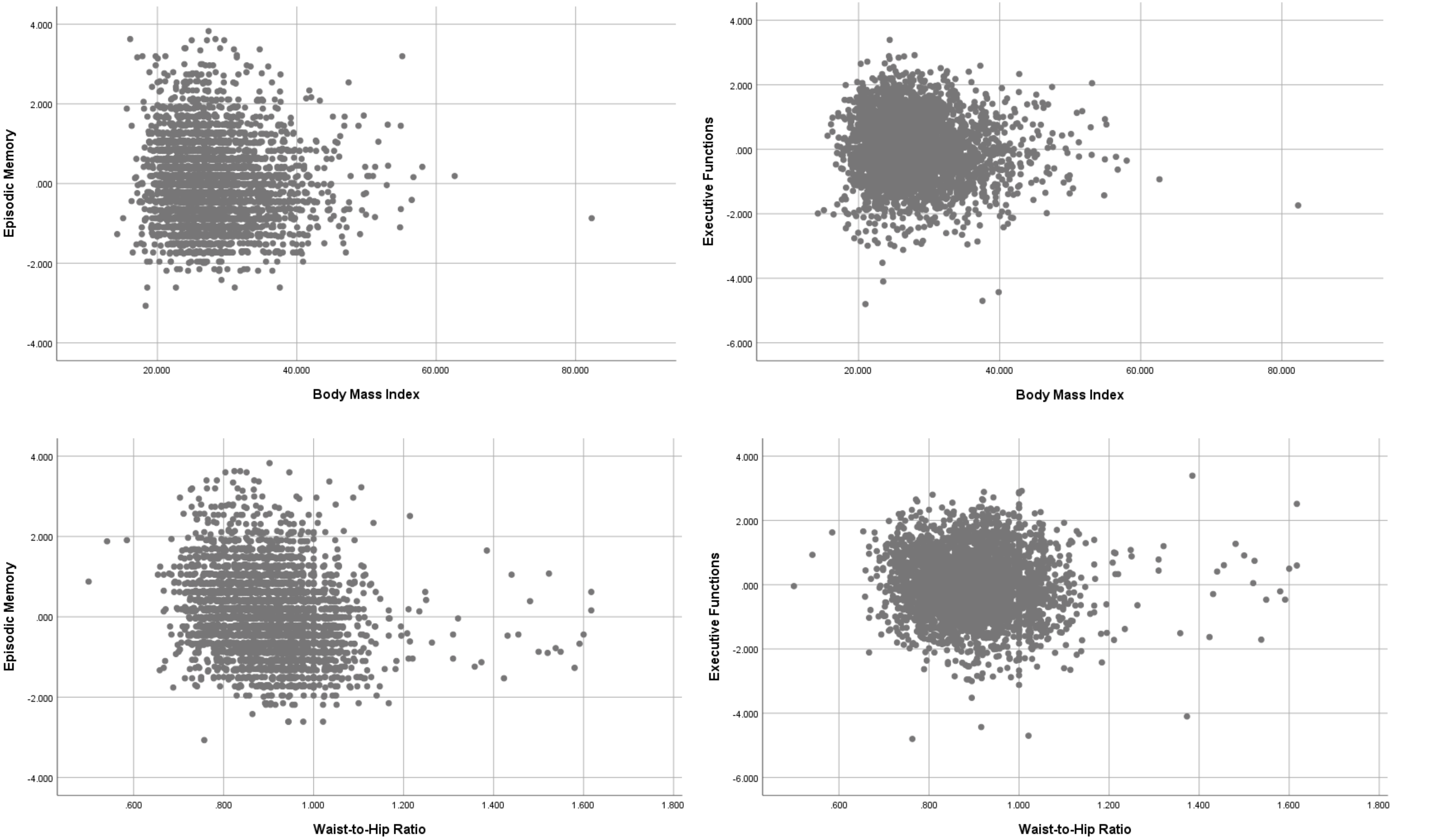

Figure B.1. Scatterplot for the unadjusted relationships between obesity and cognitive functions. The figures were presented before missing data imputation and winsorization.
